# Case report of a patient with Erdheim-Chester disease presenting with neuro-endocrine symptoms and negative for BRAF mutation

**DOI:** 10.1097/MD.0000000000033846

**Published:** 2023-05-17

**Authors:** Liuze Lu, Jing Zhou, Xu Yan, Rihua Jin, Shuanglin Deng, Weiwen Lu, Dawei Chen

**Affiliations:** a Department of Neurosurgery, The First Hospital of Jilin University, Changchun, Jilin, China; b Department of Pathology, The First Hospital of Jilin University, Changchun, Jilin, China.

**Keywords:** central nervous system, diabetes insipidus, Erdheim-Chester disease, interferon-α, negative *BRAF* mutation

## Abstract

Erdheim-Chester disease (ECD) is a rare progressive disease affecting multiple systems. It has recently been recognized as a neoplastic disease following the discovery of activating mutations in the MAPK pathway. There are several striking signs of ECD, such as the long bone involvement, as well as the hairy kidney appearance on computed tomography scan. It is rare for ECD to manifest neurological symptoms. Central nervous system involvement is a strong prognostic factor and independent predictor of death. ECD is characterized by the overproduction and accumulation of foamy histiocytes and Touton’s giant cells in various tissues and organs. ECD is a multisystem disorder in which any organ may be affected.

**Patient concerns:**

This case report describes a 57-year-old woman with headaches and ataxia as the first clinical manifestation, without characteristic bone pain, but with delayed enuresis. In addition to the renal involvement, this patient had rarer splenic involvement.

**Diagnoses:**

The imaging presentation of this patient was similar to that of a “multiple meningiomas”. A combination of clinical, imaging and pathology for the diagnosis of ECD.

**Interventions:**

Patients were given INF-α therapy.

**Outcomes:**

Fortunately, the patient responded well to INF-α treatment.

**Lessons:**

ECD patient with neuro-endocrine symptoms.

## 1. Introduction

Erdheim-Chester disease (ECD) is a rare form of non-Langerhans histiocytosis with a broad and nonspecific clinical spectrum ranging from asymptomatic to life-threatening multi-organ involvement.^[1]^ ECD was first described in 1930 by William Chester, a pathologist working in the laboratory of Jacob Erdheim. The exact incidence of ECD is unknown because of its rarity. As well as foamy histiocytes and Touton-like giant cells, ECD is characterized by fibrosis and usually affects the epiphysis and metaphyseal regions of long bones, but can affect any organ or tissue.^[2]^ About 96% of ECD cases involve bone.^[3]^

Common diagnostic methods include imaging, including brain magnetic resonance imaging (MRI), tissue biopsy, and immunohistochemistry. Their immunoreactivity includes CD68, CD163, factor XIIIa, and fascin, whereas CD1a and CD207 are negative, but their staining for S100 protein is variable.^[4,5]^ The diagnosis of ECD is made by histology and histocyte phenotyping in the appropriate clinical and radiological setting. Historically, IFN-α has been used widely to treat ECD with variable efficacy. It is rare for the CNS to be involved, and most cases are found in the context of systemic disease with intracranial lesions.^[6]^ ECD with CNS involvement is associated with a poor prognosis. ECD in the nervous system can be confused with diseases such as progressive multiple sclerosis, neurosarcoidosis, CNS vasculitis, IgG4 disease, and adrenoleukodystrophy. The diagnosis of different conditions must be ruled out with an accurate medical and clinical evaluation. ECD needs to be differentiated from the diseases listed in Table 1.

**Table 1:** Multisystem organ disease presenting as multiple infiltrative lesions of CNS.

Entity	Systemic manifestations	Radiological features	Histopathology	Treatment
CNS-LYG^[7]^	Central nervous system and systemic involvement (e.g., lung, skin, etc), respiratory and focal neurological deficits	Diffuse infiltrative intracranial lesions and mass-like lesions. Linear, patchy, nodular, mass-like, irregular ring-like enhancement.	Polymorphic lymphocytic infiltration, lymphocytic vasculitis, lymphocytic infiltration across the vessel wall, “granulomatous” lesions. CD68 (+), CD3 (+), CD20 (+).	Surgery, observation, steroid hormones, interferon, radiotherapy, rituximab, stem cell and bone marrow transplantation.
CNS-ECD^[8]^	Central nervous system with systemic involvement (e.g., bones, lungs, etc) Bone pains, urinary tears, cerebellar ataxia	Intracranial occupancy or extensive nodules involving the dura mater with a meningioma-like mass. Homogeneous enhancement.	Lipid-rich foamy cells or eosinophilic cytoplasmic histiocytes and fibroblasts. CD68 (+), CD1a (−), S-100 (+/−), Birbeck granules absent.	Surgery, observation, steroid hormones, interferon, cytotoxic drugs, radiotherapy, targeted drugs.
CNS-T-LYP^[9]^	Central nervous system and systemic involvement (e.g., lungs, joints, etc)	Multiple intracranial occupying and diffuse lesions with punctate, mass and circumferential enhancement with peripheral edema.	There is a diffuse infiltration of numerous small lymphocytes with perivascular lymphoid set phenomena and abundant reticulate fibers, with no obvious nuclear fission phase.CD3, CD4 (+), CD8 (+/−).	Surgery, steroid hormones, immunosuppressants.
CNS-IgG4-RD^[10]^	Central nervous system and systemic involvement (e.g., pancreas, salivary glands, kidneys, lungs)	Periorbital pseudotumor, pituitary inflammation, diffuse intracranial dural/dural thickening or masses.	Large lymphocytic and plasma cell infiltrate with fibrosis; IgG4 (+) plasma cells/IgG (+) plasma cells > 40%.	Steroid hormones, rituximab, immunosuppressants such as azathioprine, imatinib, and trastuzumab.
CNS-RDD^[11]^	Central nervous system with peripheral lymph node enlargement, visual changes, pituitary dysfunction, spinal cord dysfunction.	Multiple intracranial/spinal cord occupancies, meningioma-like. Uniform enhancement may have cystic changes.	Infiltration of multiple cellular components with numerous Russell vesicles visible, emperipolesis phenomenon. S-100, CD68, CD163, CD1a (+).	Surgery, observation, steroid hormones, radiotherapy, cytotoxic drugs, melphalan, immunosuppressants.

CNS-T-LYP = central nervous system T-cell lymphocyte proliferative, ECD = Erdheim-Chester disease, IgG4-RD = IgG4-related disease, LYG = lymphomatoid granulomatosis, RDD = Rosai-Dorfman disease.

## 2. Case presentation

After a month of headaches, unsteady gait, and slurred speech, a 57-year-old female was admitted to the hospital on August 2, 2021. There was no bone pain, proptosis, cough, chest tightness, or palpitations during the course of the illness. The patient had undergone a hysterectomy and adnexal resection 5 years previously. Physical examination was normal, with normal skin and mucous membranes throughout the body and no enlargement of superficial lymph nodes. During the examination, the patient presented with clear consciousness, clumsy speech, horizontal nystagmus visible on the left side of the gaze, unstable left finger-nose test, heel-knee-shin test, unstable walking, wide gait base and Romberg sign (+).

**Figure 1.**
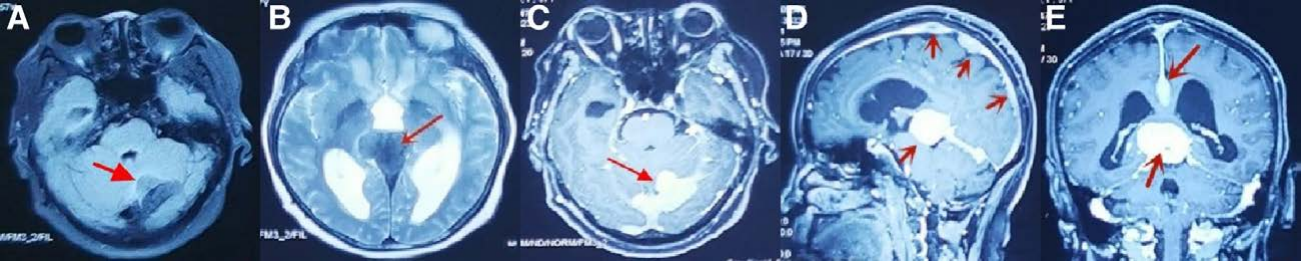
Left cerebellar curtain, pineal region lesion with low and slightly low signal on T1WI, T2WI (A, B), contrast-enhanced: diffuse striated, nodular, mass-like homogeneous enhancing lesions with well-defined borders and wide basal connection to adjacent dura in frontal, parietal and occipital pars falcae, left cerebellar curtain and pineal region (C–E).

Laboratory examination: Routine blood and urine, liver and kidney function, coagulation, thyroid hormones, and pituitary hormones were normal. Female tumor markers were negative. Computed tomography (CT) chest examination suggested limited emphysema in both lungs. Echocardiogram was normal. Abdominal CT showed mild hydronephrosis of the left renal pelvis and normal ureters bilaterally without the “hairy kidney” sign.99Tcm-MDP whole-body bone imaging did not show any radioactive concentrations. MRI shows a 3.2 × 2.8 × 2.0 cm lesion in the left cerebellar curtain area convex to the cerebellar hemisphere and a 3.7 × 4.5 × 3.0 cm lesion in the pineal gland area convex to the 3 ventricles, with low and slightly low signal in T1WI and T2WI (Fig. 1A and B). It can also be seen that the posterior part of the 3 ventricles and the midbrain aqueduct are compressed, and the supratentorial ventricles are dilated. On contrast-enhanced MRI of the head, diffuse striated, nodular, mass-like homogeneous enhancing lesions with well-defined borders and a wide base connected to the adjacent dura are seen in the frontal, parietal, and occipital pars falcao, the left cerebellar curtain and the pineal region (Fig. 1C–E).

**Figure 2.**
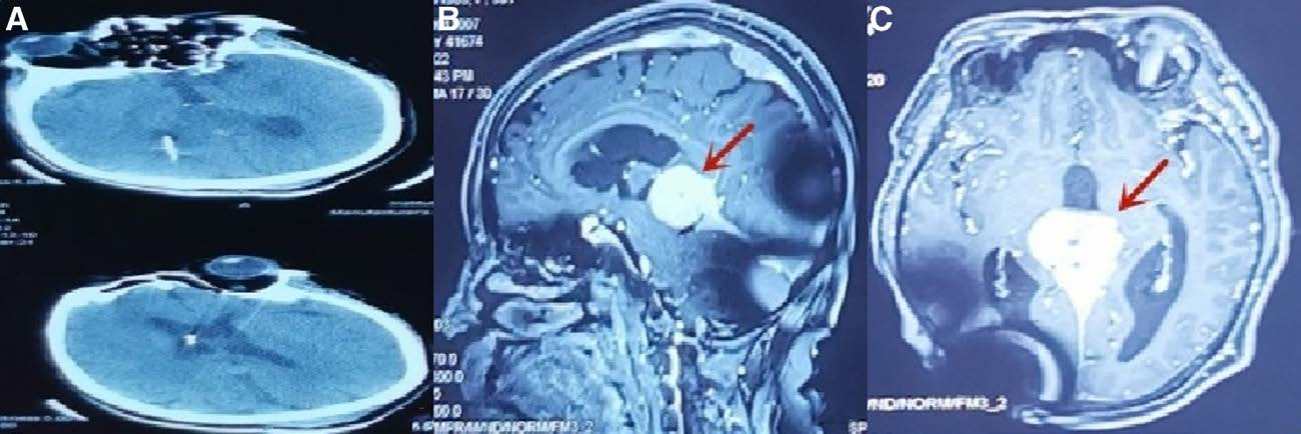
The hydrocephalus disappeared after V-P shunt (A). 5 months after V-P shunt, the lesions in the left cerebellar curtain and pineal region increased in size (B, C).

**Figure 3.**
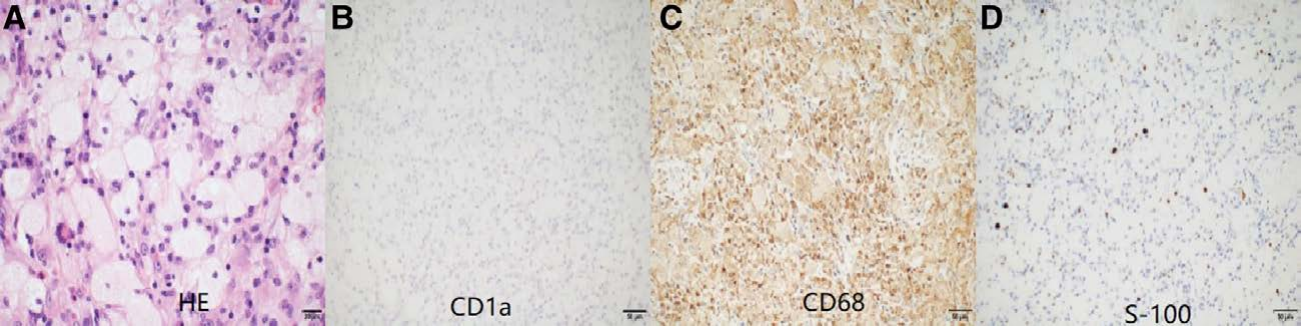
Postoperative histopathology, (A) massive foamy histiocyte infiltration with multinucleated giant cell reaction in collagenous fibrous tissue (HE, ×400), (B) CD1a-(×200), (C) CD68+(×200), and (D) S-100-(×200). Immunohistochemical staining was performed with a 2-step EnVision method.

After admission, the patient and family requested a V-P Shunt and fortunately, the patient’s clinical symptoms improved significantly after the procedure and the hydrocephalus disappeared (Fig. 2A). A recurrence of unsteady gait and enuresis (4500 mL of urine in 24 hours) 5 months later led to the patient’s hospitalization. A readmission MRI showed an enlarged lesion in the left cerebellar curtain and pineal region (Fig. 2B and C). The patient was operated on January 5, 2022, under general anesthesia. Intraoperatively, the lesion was seen to be grayish-white in color, hard and tough in texture, with a rich blood supply and tight adhesions to the cerebellar hemispheres on the left side of the cerebellar curtain. We then completely resected the lesion and the involved cerebellar curtain. The lesion in the pineal region was fused to the internal cerebral vein, the great cerebral vein and the rectus sinus, and the procedure was concluded with the delivery of several lesions for biopsy by forceps. Postoperative histopathology: a large infiltration of foamy histiocytes with multinucleated giant cell reaction was seen in the collagenous fibrous tissue (Fig. 3A). Immunohistochemistry: KI-67 (−), P53 (−), GFAP (−), Oligo-2 (−), NeuN (−), PR (−), SSTR2 (−), EMA (−), CD34 (−), Langerin (−), Lysozyme (+), Vimentin (+), CyclinD1 (scattered +), ALK (−), CD1a- (Fig. 3B), CD68 + (Fig. 3C), S100- (Fig. 3D). No Birbeck granules were seen on electron microscopy. *BRAF* V600E, *NRAS*/*KRAS* exons 2, 3 and 4, negative for *PIK3CA* mutation gene. Diagnosis: Erdheim-Chester disease.

**Figure 4.**
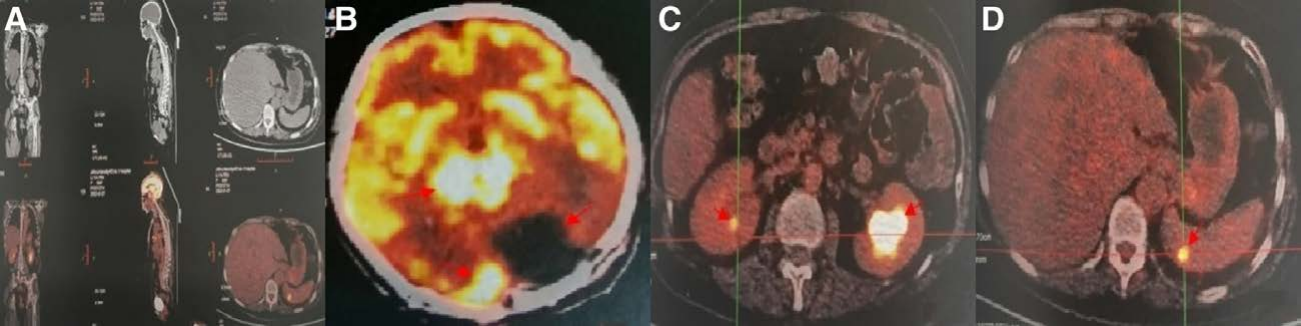
PET/CT showed normal distribution of skeletal radioactivity throughout the body (A), no abnormal radioactive uptake in the left cerebellar curtain region and hypermetabolic foci in the falx and pineal regions (B). angiosmooth muscle lipoma in the right kidney, hydronephrosis in the left kidney (C) and hypermetabolic nodules in the spleen (D). PET/CT = positron emission tomography/computed tomography.

The patient had no surgical complications and continued desmopressin acetate 0.05mg (1 time/8 hours) orally for 10 days, and urine output decreased to 1800 mL/day. IFN-α was administered 600 mIU/dose 3 times/week, and the patient was discharged after 1 month of treatment with a normal gait. IFN-a was gradually increased to 900 mIU/dose 3 times/week according to the patient’s tolerance at the local hospital, and the patient discontinued INF-a at the 6th month of treatment due to fever and muscle pain. FDG- Positron emission tomography and CT examination of the whole-body skeleton showed normal results of radioactivity distribution (Fig. 4A), no abnormal radioactivity uptake in the left cerebellar curtain area, hypermetabolic foci in the falx and pineal regions (SUV max 16.3, Fig. 4B). Vascular smooth muscle lipoma in the right kidney, hydronephrosis in the left kidney (Fig. 4C), and hypermetabolic nodules in the spleen (SUV max 7.5, Fig. 4D). We followed up with the patient 8 months after discharge with no new clinical symptoms and normal life.

## 3. Discussion

This patient started with high cranial pressure and cerebellar dysfunction without the most common characteristic bone pain. Parenchymal CNS lesions are an important cause of ECD dysfunction. The patient presented 5 months after the initial visit with uveitis, and the MRI showed a mixed pattern resembling a “multiple meningiomas.” It has been reported that patients with CNS-ECD can have symptoms that are similar to meningiomas, granulomatous diseases, or meningeal infiltrates with Rosai-Dorfman disease and LCH. It is generally not seen in the parenchyma of the brain that fibroplasia occurs, unlike in the meninges and other tissues involved in ECD. There was mild hydronephrosis of one renal pelvis and no “hairy kidney” sign.

There is no standard treatment for ECD. Baseline imaging to determine the extent of the disease, biopsy to confirm characteristic histopathology, and analysis to assess *BRAF* V600E mutation status are essential before starting treatment. Patients with *BRAF* V600E mutation can be treated with the BRAF inhibitor vemurafenib. Patients with *BRAF* wild type may be treated with high-dose interferon, while those with CNS-ECD with local compression should have the compression surgically removed and the pathology clarified. In 8 cases of severe disease (including CNS and cardiac involvement), “standard” doses of IFN-α were ineffective, while higher doses of IFN-α (IFN-α > 18 mIU/week) were more effective.^[12]^ There are several potential toxicities associated with IFN-α, including fever, fatigue, flu-like symptoms, myalgia and arthralgia, neuropsychiatric and gastrointestinal symptoms, alopecia, pruritus, transaminitis, and myelosuppression. There were no significant differences in side effects between IFN-α dose levels.^[13]^ In recent years, drugs such as Vemurafenib, Anakinra, infliximab, tocilizumab, Cladribine, cobimetinib, Mektovi, and Rapamycin have brought new hope to patients with multisystem or refractory ECD, but BRAF, MEK, cytokines, the mTOR inhibitors, and cytotoxic drugs still require large case summaries and validation for treatment duration, resistance, toxicities, maintenance doses and withdrawal, and radiation therapy is still controversial.^[14]^

## 4. Conclusion

This case report highlights the importance of considering ECD in the differential diagnosis when patients present with central nervous system symptoms of unknown etiology. Neurologists play a key role in the identification and follow-up of ECD and other histiocytic diseases, as patients may present with neurological symptoms and develop isolated neurological disorders, and neurological involvement is associated with a poorer prognosis. Neurological manifestations are often the only symptoms patients with ECD experience in the early stages of the disease. Therefore, when treating patients with new onset seizures, ataxia, or cognitive difficulties, it is important to keep in mind ECD if intracranial parenchymal or dural masses are found on cranial imaging. In addition, it emphasizes the importance of biopsy for cytopathology and IHC testing to determine the correct treatment plan. A histologic examination is crucial for the final diagnosis, and close, serial follow-up after surgical resection is necessary to identify recurrence and progression.

## Acknowledgments

This study was supported by a grant from the Department of Science and Technology of Jilin Province (20210401137YY). The authors would like to thank Medjaden Bioscience Limited (https://www.medjaden.com/show-790.html) for the English language editing and review services.

## Author contributions

**Conceptualization:** Liuze Lu, Jing Zhou.

**Data curation:** Liuze Lu, Rihua Jin.

**Formal analysis:** Xu Yan, Rihua Jin, Shuanglin Deng, Dawei Chen.

**Funding acquisition:** Jing Zhou, Shuanglin Deng, Weiwen Lu.

**Project administration:** Weiwen Lu, Dawei Chen.

**Resources:** Jing Zhou.

**Supervision:** Xu Yan, Rihua Jin, Dawei Chen.

**Software:** Shuanglin Deng.

**Validation:** Liuze Lu.

**Visualization:** Liuze Lu, Xu Yan, Weiwen Lu.

**Writing – original draft:** Liuze Lu, Dawei Chen.
